# Tremor modulation: a systematic review of resetting by single-pulse transcranial magnetic stimulation

**DOI:** 10.55730/1300-0144.6175

**Published:** 2026-01-23

**Authors:** Handan UZUNÇAKMAK-UYANIK, Çağrı Mesut TEMUÇİN

**Affiliations:** 1 Institute of Neurological Sciences and Psychiatry, Hacettepe University, Ankara, Turkiye; 2 Department of Neurology, Faculty of Medicine, Hacettepe University, Ankara, Turkiye

**Keywords:** Phase resetting, systematic review, transcranial magnetic stimulation, tremor, tremor resetting

## Abstract

**Background/aim:**

Tremor arises from mechanical, reflex, or central oscillatory mechanisms. Transcranial magnetic stimulation (TMS) can transiently perturb ongoing tremor and enables the quantitative assessment of phase resetting, offering circuit-level insight into tremor types. Although numerous studies have applied TMS-induced resetting, the findings have not been systematically reviewed. This study systematically reviews human studies evaluating TMS-induced tremor resetting across tremor types.

**Materials and methods:**

A systematic search of PubMed and Google Scholar identified human studies using TMS to perturb tremor or rhythmic movement. Search terms included “tremor resetting,” “resetting of tremor,” “tremor phase shift,” “tremor phase reset,” “transcranial magnetic stimulation,” and “central oscillator.” Inclusion criteria were human participants, experimental TMS perturbation, and quantitative tremor phase/resetting outcomes. Exclusion criteria were animal studies, therapeutic repetitive TMS trials without resetting analyses, and isolated case reports. Two researchers independently screened and extracted data. The PRISMA 2020 guidelines were followed.

**Results:**

Twenty-one studies were identified, three of which were excluded from the primary synthesis (two case reports and one qualitative-only design). Eighteen studies remained, which addressed essential tremor (ET) (n = 6), Parkinson’s disease tremor (PDT) (n = 7), orthostatic tremor (OT) (n = 4), palatal tremor (n = 1), dystonic tremor (DT) (n = 1), and voluntary rhythmic movement (n = 4). M1 stimulation reset ET, postural PDT, OT, palatal tremor, DT, and voluntary rhythmic movements. Rest PDT had inconsistent resetting by M1 stimulation and no resetting by cerebellar stimulation. Cerebellar stimulation reset postural PDT but not ET. The resetting index was associated with the stimulus intensity and duration of the silent period.

**Conclusion:**

TMS-induced resetting is a strong physiological tool for differentiating tremor circuits. M1 acts as a major convergence node, while cerebellar involvement is tremor-specific. Methodological heterogeneity and small samples limit the comparability of study results. Advances in targeting technologies and closed-loop and phase-locked protocols could enhance the diagnostic and therapeutic utility of resetting paradigms.

## 1. Introduction

Tremor is an involuntary, rhythmic oscillation that has four main mechanisms proposed to account for its emergence: the mechanical properties of the body segment, stretch-reflex contributions from the extremity, intrinsic oscillatory tendencies of neurons in specific brain regions, and rhythmic activity arising when cerebellar feed-forward or feedback control loops become unstable [1]. Central tremor oscillatory typically results from the interaction of abnormal neural activity between several neural pathways such as the cerebello-thalamo-cortical (CTC) and basal ganglia-thalamo-cortical (BGTC) circuits. It is the movement disorder that has been investigated most frequently to date using neurophysiological methods. The primary reason for this is the need to differentiate tremor types and to elucidate the underlying pathophysiology.

One method for identifying the effects of various inputs on the central oscillator of the tremor is phase resetting, a technique originally applied for tremor but in principle suitable for any rhythmic behavior [2]. The resetting technique is used to distinguish whether a tremor originates from a central oscillator or arises from peripheral mechanical feedback. If resetting is induced by a central stimulus, the tremor likely originates from a central oscillator. Conversely, if the rhythm continues unchanged following a central stimulus, the tremor is more likely driven by peripheral mechanical feedback mechanisms, such as the muscle spindle and/or Golgi tendon organ reflex circuitry. In recent years, transcranial magnetic stimulation (TMS) has become the predominant method for investigating tremor resetting, while some other studies used peripheral mechanical stimuli and transcranial alternating current stimulation. TMS is a noninvasive and painless method for the investigation of human brain function. It generates a magnetic field and allows the assessment of neural circuits involved in motor control and movement disorders, such as BGTC and CTC circuits [3]. This makes it a valuable tool for the study of the central components of tremors.

Over the past three decades, TMS-induced tremor resetting has been investigated for several tremor types, including essential tremor (ET), Parkinson’s disease tremor (PDT), orthostatic tremor (OT), palatal tremor, dystonic tremor (DT), and voluntarily mimicked tremor/rhythmic movements. It should be noted that voluntarily mimicked tremor and voluntary rhythmic movements should not be regarded as pathological oscillators. Instead, they serve as physiological control models that allow systematic investigation of timing mechanisms, cortical excitability, and phase resetting behavior in the absence of pathological oscillators. The findings of relevant studies remain scattered, derive from small samples, and employ heterogeneous stimulation parameters and analysis methods. To date, no systematic synthesis has integrated these threads of literature to determine which tremor types can be reset by TMS, which neural targets mediate resetting, and how methodological differences influence the results. Therefore, the aim of this systematic review is to identify, evaluate, and synthesize all human studies that have examined TMS-induced resetting in tremor types using standardized eligibility criteria and structured data extraction in accordance with the PRISMA 2020 guidelines [4].

## 2. Materials and methods

### 2.1. Search strategy

The literature search was conducted with PubMed and Google Scholar using the following keyword phrases: “tremor resetting,” “resetting of tremor,” “tremor phase shift,” “tremor phase reset,” “transcranial magnetic stimulation,” and “central oscillator.” The search was performed in November 2025 and limited to English-language articles published from 1980 to 2023. Reference lists of all identified studies and relevant review articles were also screened for more eligible publications to include in the review.

### 2.2. Eligibility criteria

Original research articles were included if they:

1. were peer-reviewed,
2. involved human participants,
3. used TMS as an experimental perturbation to examine tremor or voluntary rhythmic movement, and
4. reported quantitative results related to phase resetting or phase shift.

Studies were excluded if they:

1. involved animals,
2. were case reports,
3. were paired-pulse TMS or therapeutic repetitive TMS trials without phase-resetting analysis,
4. only realigned qualitatively without measurable phase-based metrics, or
5. were review papers.

### 2.3. Study selection

The initial search yielded 74 results. Titles and abstracts were screened for potential inclusion. Full-text screening was done for all articles that passed that first stage. Two independent researchers (H.U.U. and Ç.M.T.) checked each study against the criteria listed above, and any differences in decisions were resolved through discussion.

### 2.4. Data extraction

Information on tremor type, number of subjects, site/region of stimulation, TMS parameters, recorded muscles, method of analysis, and whether or not tremor resetting with TMS was present was recorded for each of the included studies.

### 2.5. Risk of bias assessment

The assessment of methodological quality and risk of bias was done using the Joanna Briggs Institute (JBI) Critical Appraisal Checklist for Analytical Cross-Sectional Studies. Each study that was selected for the review was assessed in terms of the eight JBI criteria and scored as “Yes,” “No,” “Unclear,” or “Not Applicable.” In this evaluation, special attention was paid to participant descriptions, clarity of stimulation parameters, electromyography (EMG)/accelerometry signal processing, transparency of analysis methods, and completeness of outcome reporting.

The JBI Critical Appraisal Checklist for Analytical Cross-Sectional Studies was selected because the majority of the included studies were experimental human neurophysiology studies with cross-sectional designs, in which exposure (TMS perturbation) and outcome (tremor phase resetting) were assessed within the same experimental session. Alternative risk-of-bias tools developed primarily for interventional or longitudinal studies were considered less suitable for capturing the methodological features of these short-duration, physiology-based experiments.

### 2.6. PRISMA reporting

This systematic review was conducted and reported in accordance with the PRISMA 2020 guidelines [4].

## 3. Results

### 3.1. Study selection

The initial literature search performed with PubMed and Google Scholar yielded 101 records. A total of 21 full-text articles were selected for eligibility assessment after the removal of duplicates and the screening of titles and abstracts. Of these, three were excluded from the primary analysis (two single-patient case reports and one study in which only qualitative burst realignment without quantitative phase-resetting analysis was used). Eighteen studies remained for the main systematic synthesis. The PRISMA flow diagram (Figure) depicts the study selection process.

### 3.2. Study characteristics

All included studies were human experimental neurophysiology investigations in which single-pulse TMS (spTMS) was used to perturb ongoing tremor or voluntary rhythmic movement and the consequent tremor resetting was quantified. Most experiments targeted the contralateral primary motor cortex (M1), a few additionally stimulated the supplementary motor area (SMA) or cerebellum, and some studied bilateral or multiple cortical targets [2,3,5–15]. The studied tremor types were ET, PDT (rest, postural, and reemergent), OT, PT, DT, and voluntarily mimicked tremor/voluntary rhythmic movement.

In the selected studies, spTMS intensity was typically set as a percentage of the resting motor threshold. Some protocols systematically varied the intensity to characterize its impact on resetting. Tremor or movement was recorded by EMG, usually from the most affected limb or relevant muscles. The main design features and outcome measures of all selected studies are summarized in Tables 1 and 2. Although several studies also reported paired-pulse or repetitive TMS paradigms, only data from the studies that used spTMS as an acute perturbation tool for tremor resetting analysis were considered.

### 3.3. Risk of bias in included studies

Risk of bias was evaluated using the JBI Critical Appraisal Checklist for Analytical Cross-Sectional Studies. Most of the studies clearly described the inclusion criteria, participant characteristics, stimulation parameters, and outcome measurement and had consistent “Yes” ratings for these domains. Variability was observed mainly in the identification and handling of potential confounders, for which some studies were rated as “Unclear” or “Not Applicable,” reflecting the experimental neurophysiology design of these protocols. In general, the methodological quality of the studies was acceptable and no study demonstrated major risk-of-bias issues that could hinder interpretability. Detailed assessment results for risk of bias for all included studies are provided in the Supplementary Table.

### 3.4. Study findings

#### 3.4.1. Concept and quantification of tremor resetting

The fundamental principle of resetting is to determine whether an input can shift the oscillator to a specific phase within its cycle. If such a reset occurs, it is concluded that the input has a significant influence on the timing of the oscillator [2]. Biological oscillators exhibit phase-resetting behavior and, based on that, they are represented as nonlinear limit-cycle oscillators whose phase can shift to a new steady-state if a sufficiently strong and sudden perturbation is applied [16]. If the stimulus is very weak, no reset occurs. Additionally, it will not reset if the stimulus does not reach the oscillator effectively or if the oscillator is inherently too stable. When the new phase is plotted against the prestimulus phase, complete phase resetting appears as a slope of 0 (type 0 resetting), whereas no resetting produces a slope of 1 (type 1 resetting) [16]. Resetting studies assume that if a TMS pulse disrupts or realigns the tremor rhythm, the stimulated region should participate in generating or sustaining that tremor.

The resetting index (RI) is defined as the slope of the linear regression relating poststimulus phase shifts to the prestimulus phase and it is used in many tremor resetting studies [2,3,6–8,10,11,17–19]. It is derived from the changes in the phase of the cycle during repeated stimulations and shows the pattern of the estimated poststimulus phase changes as a function of the oscillator’s initial phase [2]. Because biological phases are inherently unstable immediately after stimulation and they also show cumulative cycle-to-cycle variability, the RI acts as a statistical description of these noisy phase relations rather than a direct measure of the oscillator’s physiological response [16]. It causes phase-shift plots to deviate from the idealized unity slope even if there is actual resetting. Correct interpretation of phase-resetting requires recognition that the RI does not quantify the “amount” of resetting. RI value of 0.6 does not indicate that the tremor is “60% reset”; rather, it shows the degree of confidence with which one can infer that resetting occurred. This metric only reflects the strength of the statistical association between old and new phase estimates and, hence, the certainty that true type 0 phase resetting is occurring.

#### 3.4.2. Single-pulse TMS and tremor

Processes involving spTMS, which largely activates corticospinal neurons transsynaptically [20], typically apply a single stimulus to the primary motor cortex (M1) to probe corticospinal excitability, assessed through motor evoked potentials (MEPs), rest and active motor thresholds (RMT and AMT), and the cortical silent period (SP) [1]. While MEPs and the thresholds index motor cortex excitability, the SP, mainly mediated by cortical GABAergic inhibition, reflects inhibitory circuit function, with its shortening indicating impaired inhibition [1].

Several studies have examined spTMS measures in ET: no differences in SP duration between ET and healthy control (HC) groups were reported [21], no differences in RMT or SP were found between ET and HC groups [22], RMT did not differ between ET and HC groups while motor imagery-induced MEP facilitation increased in the HC but not in the ET group [23], lower RMT and AMT values in the ET group compared to the HC group were described [24], and no differences in MEP amplitudes between ET and HC groups were observed [5]. Additionally, in a study in which shoulder position was incorporated into the experimental design, MEPs were smaller at rest but larger upon moving the shoulder to 30° horizontal abduction in the HC and ET groups. For PTD patients, MEPs were lower during activation upon moving the shoulder to 30° horizontal abduction [25].

spTMS paradigms have targeted various brain regions, primarily including M1 and the cerebellum, to determine which circuits act as the primary oscillator and which are responsible for modulating existing tremor across different tremor types [5–8, 17, 26]. Thus, spTMS paradigms enable investigators to perturb cortical activity and observe its influence on ongoing tremor [27]. This phenomenon is referred to as tremor resetting. Whether such resetting is transient or steady-state has also been examined in some studies [5, 6, 9, 28].

#### 3.4.3. Tremor type-specific findings on tremor resetting

##### 3.4.3.1. Essential tremor and resetting

In cases of ET, spTMS delivered to the motor cortex has been shown to reset the tremor [3, 5, 6, 11]. However, in some studies, the same spTMS failed to reset the tremor when applied to the cerebellum [3, 5]. It was proposed that the distal thalamo-cortical portion of the CTC circuit may contribute more strongly to tremor generation than the proximal cerebello-thalamic portion, which could explain why motor-cortical stimulation produced resetting whereas cerebellar stimulation did not [3].

Hellriegel et al. [29] approached studies demonstrating ET resetting with spTMS over M1 from a different perspective, arguing that the suprathreshold intensities used in these experiments affect the entire motor system, including its cortical, subcortical, spinal, and even peripheral components, and therefore leave unresolved the question of which level of the motor system is actually responsible for TMS-induced tremor resetting.

Lu et al. [3] found that ET postural tremor was reset by TMS stimulation from both M1 and SMA. This means that SMA also plays an active role in ET pathophysiology. However, they found no difference in RI values between M1 and SMA in cases of postural ET. They attempted to interpret the lack of tremor modulation after cerebellar TMS in ET and proposed two possible explanations. One explanation is that the cerebellar nuclei implicated in tremor generation are located too deeply for the TMS-induced current to sufficiently influence their activity. Another is that ET pathology may engage the cerebellum bilaterally, making unilateral stimulation inadequate to produce tremor resetting [3]. Pinto et al. [5] proposed two additional explanations for the lack of tremor resetting with cerebellar stimulation in ET: first, that the abnormal cerebellar function could stem from alterations in the afferent pathways, and second, that the thalamocortical loop may serve as the oscillator in ET, making cerebellar stimulation insufficient to modify the thalamocortical oscillation. Shih and Pascual-Leone [27] also noted the possibility of inefficient coil geometry or current shunting across cerebrospinal fluid spaces that could prevent direct stimulation of motor regions.

Yu et al. [12] demonstrated that spTMS could reset ET, indicating its central origin, whereas spTMS could not reset the cardiac rhythm since the heart is controlled entirely by a peripheral pacemaker. They noted that this finding indicates that the mechanism of ET is different from that of the heart and supports the safety of using TMS as a physiological probe in ET.

Pascual-Leone et al. [6] demonstrated the relationship between resetting and SP, showing that although stimulus intensity influences the degree of resetting, the effect is most closely associated with the duration of the post-MEP SP in ET.

##### 3.4.3.2. Parkinson’s disease tremor and resetting

Some studies have examined only postural tremor [6, 11] in Parkinson’s disease, while others have examined more than one tremor type in resetting studies of Parkinson’s disease [3,8, 9, 19, 28].

Lu et al. [3] demonstrated that both spTMS and paired-pulse TMS applied over M1 modulated rest and postural tremor in Parkinson’s disease. The functional contribution of M1 to PDT resetting was also supported by other spTMS studies [6, 8]. However, it was shown that rest PDT was not reset by either spTMS or paired-pulse TMS applied over the cerebellum [3]. Ni et al. [8] showed that cerebellar TMS reset postural tremor but failed to reset rest tremor in Parkinson’s disease, implying that the CTC network contributes to postural tremor generation in these cases but not to rest tremor. Hoppenbrouwers et al. [30] suggested that the lack of tremor resetting with cerebellar stimulation in rest tremor may be related to the cerebellum’s role in fine motor control and precise calibration, functions that are not required during the resting state. Helmich et al. also observed that cerebellum TMS reset reemergent tremor but not rest tremor [28].

In the study by Britton et al. [11], TMS applied over the contralateral motor cortex was found to reset postural PDT. However, the period was significantly shortened after TMS and the latency of reappearance of rhythmic EMG activity varied according to the preexisting tremor period, unlike in ET and mimicked tremor. This finding indicated that after resetting the postural PDT restarts according to the intrinsic cycle length of its own oscillator [11]. In other words, postural PDT is reset, but the oscillator reestablishes its phase based on its inherent cycle duration.

In a study aiming to understand the mechanism of PDT suppression, defined as the brief disappearance of tremor in some patients with Parkinson’s disease with rest tremor during the transition from rest to a posture-holding position, no significant differences were found in RI or resetting stability (RS) values for rest tremor between patients with and without tremor suppression (RI: 2.1 ± 0.6 vs. 1.8 ± 0.5, t = 1.59, p = 0.12; RS: 0.99 ± 0.3 vs. 0.9 ± 0.4, t = 0.66, p = 0.516) [19]. In contrast, patients with tremor suppression had significantly higher postural tremor RI values (2.0 ± 0.3 vs. 1.4 ± 0.7, t = 2.56, p = 0.020) and RS values (1.0 ± 0.2 vs. 0.7 ± 0.3, t = 3.20, p = 0.005) compared to those without tremor suppression [19]. When evaluated together with the other parameters of that study, these findings led to the inference that the mechanism of postural tremor may differ between patients with and without tremor suppression [19].

Ni et al. [8] demonstrated that rest PDT can also be reset by M1 stimulation and they noted that this had not been previously reported. This finding suggested that M1 may also play a role in the generation of rest PDT. They also reported that M1 stimulation produced a greater degree of resetting in postural PDT compared to rest PDT.

Leodori et al. [9] reported that TMS applied over M1 induced a significant and steady-state resetting for both reemergent and rest tremor. The average RI values were 2.07 ± 0.10 (t = 12.352, p < 0.001) and 2.16 ± 0.17 (t = 20.387, p < 0.001), respectively. A paired t-test showed nonsignificant differences between reemergent and rest tremor for both RI (t = 0.70, p = 0.504) and the RI1/RI5 ratio (t = 0.58, p = 0.574).

##### 3.4.3.3. Orthostatic tremor and resetting

In the study by Tsai et al. [7], two patients with primary OT had mean RI values of 0.93 and 0.82, demonstrating that a central oscillator involving the motor cortex plays an important role in the generation or modulation of OT. They further stated that if a tremor does not respond to brain stimulation, a subcortical origin for the tremor becomes more likely.

Tsai et al. [7] also observed that OT can be reset by spTMS. In contrast to this previous study, which examined only the effect on the contralateral leg, Spiegel et al. [13] evaluated the impact of stimulation on both ipsi- and contralateral leg muscles. They found that resetting occurred similarly in both the ipsi- and contralateral leg muscles and the resetting threshold was higher than the motor threshold. However, in their study, the classical RI method of Lee and Stein [18] was not used; instead, the “stimulus-induced shift” measured in milliseconds was taken as the indicator of resetting [13]. On the other hand, Mills and Nithi [31] reported no resetting effect with TMS over the motor cortex in patients with OT. However, they used lower stimulus intensities. Furthermore, Mills and Nithi [31] suggested that the motor cortex is neither the site nor the regulator of the presumed central generator of primary OT and that the other intracortical structures activated by TMS also do not modulate the oscillator [31].

##### 3.4.3.4. Other tremor types and resetting

###### 3.4.3.4.1. Palatal tremor and resetting

In the study by Chen et al. [14], the mean motor threshold (MT) was 44 ± 4% in patients with symptomatic palatal tremor (n = 5), and when the stimulus intensity was set at 200 % MT, the RI values ranged from 0.86 to 0.96. At higher stimulus intensities, RI values were higher and the tremor resetting persisted for a longer duration. Patients with lower MTs exhibited higher RI values [14]. In addition, the average latency of tremor reappearance after TMS was markedly prolonged compared to the average prestimulus interval and the degree of resetting was strongly correlated with stimulus intensity and the latency of tremor reappearance after TMS [14]. These authors concluded that the degree of resetting was not related to the timing of the stimulus within the tremor cycle.

###### 3.4.3.4.2. Dystonic tremor and resetting

Pattamon et al. [32] showed that M1 stimulation can also reset tremor in DT, whereas cerebellar stimulation produced a more pronounced tremor resetting in ET than in DT. This pattern suggested that ET and DT involve different oscillatory pathways [32].

##### 3.4.3.5. Voluntarily mimicked tremor/voluntary rhythmic movements and resetting

In the study by Britton et al. [11], mimicked tremors were examined with frequencies of 4.0 to 6.5 Hz [11]. The time to reappearance of rhythmic EMG activity following spTMS occurred at a fixed latency in cases of mimicked tremors. Additionally, the period of mimicked tremors after spTMS did not change.

Wagener and Colebatch [2] showed in their study, in which nine sites covering a 5-cm square region of the contralateral cortex were systematically stimulated, that magnetic stimulation of the contralateral motor cortex was most effective in resetting voluntary movement. They noted that the relatively slow movement rate chosen by their participants, which was slower than that of most tremors, made it possible to clearly identify the events that occurred after the magnetic stimulus.

Wagener and Colebatch used “nett resetting” as a measure of phase resetting, based on the relative amplitudes of the averages of the stimulated and a phase-locked control position record [2]. An approximately linear relationship was observed between “nett resetting” and the RI value, accompanied by a fitted regression trend [2]. At cortical sites where spTMS produced resetting, EMG responses showed an initial brief excitation, followed by an inhibitory SP, and then a short synchronous burst in both wrist flexors and extensors before the usual alternating rhythmic activity resumed [2]. Additionally, Wagener and Colebatch [2] stated that the timing of the first rhythmic EMG peak after the stimulus showed a strong correspondence with the cycle duration of the subject’ prestimulus movement. This finding implies that this factor must also be considered when analyzing the RI across different tremor types, given that the SP has a strong influence on the RI.

TMS is known to delay the onset of voluntary ballistic movements as a motor cortex-related phenomenon [33]. This delay typically ranges between 80 and 150 ms in voluntary ballistic movements [33], which is shorter than the SP reported by Wagener and Colebatch (of the order of 400 ms for some participants with voluntary rhythmic movements) [2]. A plausible explanation for this discrepancy as suggested by Wagener and Colebatch [2] is that the “time keeper” is reset and requires some time after the cortical shock to resume its activity, but this activity is masked by the SP.

By keeping the TMS intensity constant throughout their experiment, Wagener and Colebatch [15] showed that the magnitude of the applied extension torque had a strong influence on both the RI and the null phase, with the largest torque producing the highest RI values (mean RI: 0.72). They further argued that the magnetic pulse was unlikely to reset voluntary movement through a direct stimulation on the motor pathways. Instead, they proposed an indirect mechanism: the cortical shock transiently disrupts the ongoing flexion-extension cycle, and the afferent feedback generated by the induced twitch subsequently realigns the movement timing and produces the observed resetting.

In another study, Chen et al. [10] reported that in unimanual tasks, TMS disrupted rhythmic movements in both the contralateral and ipsilateral hands, although the ipsilateral effect was considerably weaker. During bimanual in-phase movements, TMS simultaneously reset the rhythm of both hands relative to unimanual tasks, but its influence on the contralateral hand was less, whereas the ipsilateral effect was greater. Comparable patterns emerged with stimulation of either hemisphere. In contrast, TMS had only minimal effects on bimanual antiphase movements. These authors concluded that the comparable effects of right and left hemisphere stimulation suggested no dominant motor cortex for simple bimanual in-phase coordination. The weaker contralateral and stronger ipsilateral influence during in-phase bimanual movement, relative to unimanual tasks, pointed to interhemispheric coupling [10]. The limited susceptibility of antiphase movements to TMS further indicated distinct rhythm-control mechanisms across the two coordination modes [10]. Finally, the transient desynchronization induced by TMS implied that both motor cortices operated downstream of a shared timing command or that rhythmic timing emerged from balanced bidirectional communication between the hemispheres [10].

### 3.5. Integrated assessment of TMS-induced resetting findings in tremor types

It should be noted that substantial methodological heterogeneity exists across the analyzed studies. Differences in stimulation targets, stimulus intensities, EMG acquisition protocols, and RI calculation methods limit direct quantitative comparisons between these studies. Accordingly, the findings of this review are synthesized qualitatively rather than quantitatively.

The main results reported by Britton et al. [11] in their spTMS resetting tremor study conducted with patients with PDT, patients with ET, and healthy volunteers mimicking tremor were as follows: In patients with PDT, the RI varied between 0.90 and 0.99, with a mean of 0.96 ± 0.01. A similar range was found in ET patients (0.90–0.99; mean: 0.97 ± 0.01). In healthy volunteers mimicking tremor, the values ranged from 0.68 to 0.99 with a mean of 0.91 ± 0.04. Analysis of variance (ANOVA) showed no significant differences among the group means (F = 1.88, df = 2). Stimulation over the contralateral motor cortex transiently suppressed rhythmic EMG activity in all tremor types, and the activity reappeared time-locked to the pulse [11]. In postural PDT, the latency of this reappearance varied with the proportion of the ongoing tremor period [11]. In contrast, ET and mimicked tremor reemerged at constant latencies [11]. This finding suggests that magnetic pulses delivered centrally can modulate all three rhythms but PDT had a central oscillator that reset the tremor to a fixed phase and then let it continue at its intrinsic cycle length, whereas ET and mimicked tremor did not show this oscillator-dependent timing [11]. The authors concluded that these differences support the existence of distinct underlying pathophysiological mechanisms across these tremor types [11]. On the other hand, the final conclusion of Lu et al. [3] is noteworthy. They stated that even though these two tremor types may originate in different subcortical motor circuits (i.e., the BGTC circuit in PDT vs. the CTC circuit in ET), M1 is probably a convergent stage for the final tremor output.

Pascual-Leone et al. [6], who demonstrated tremor resetting in both ET and PDT, proposed two possible mechanisms to explain the effect of motor cortex TMS. First, TMS may transiently suppress the activity of a central tremor generator located within the motor cortex, thus resulting in the resetting of the oscillation. Second, TMS-induced changes may modulate the reflex pathways of the transcortical long-loop. They further emphasized that only steady-state resetting could be considered as genuine evidence of an effect on the central oscillator.

In some studies on PDT and ET, tremor resetting was examined with the forearm held in an outstretched posture, a condition in which motor neurons are already highly activated to maintain the position [6, 11]. This volitional control and sensory feedback increase the motor cortex excitability; thus, postural tremor can be reset with a relatively lower stimulus intensity. On the other hand, facial muscles lack proprioceptors, the trigeminal system does not experience recurrent inhibition, and peripheral trigeminal nerve stimulation does not modulate tremor rhythm [34, 35] . Based on this knowledge, Chen et al. [14] stated that sensory feedback has only a minimal role in the resetting of symptomatic palatal tremor and that TMS most probably resets symptomatic palatal tremor through a central mechanism rather than a peripheral one.

Considering the interburst periods after spTMS, the interval between tremor bursts was shortened in PDT after TMS [11], and similar shortening was also observed in OT [7, 13]. In contrast, no changes were reported in ET or in voluntary rhythmic movements [11]. It was stated that the pathophysiological mechanisms underlying OT and PDT are different from those that cause ET and voluntarily mimicked tremors [7, 11]. Tsai et al. [7] also highlighted that these phenomena indicate the motor cortex’s involvement in either their generation or modulation. However, in patients with symptomatic palatal tremor, the first two cycle intervals of the ongoing tremor were significantly prolonged. Chen et al. [14] declared that this finding differs from results seen for other tremor types and the mechanism underlying this transient slowing of symptomatic palatal tremor frequency remains unclear [14]. Moreover, they stated that the close association between the resetting of PDT/ET and the TMS-induced SP [6] suggests that the modulation of tremor rhythm by TMS occurs primarily through an inhibitory mechanism [14].

Since neuroimaging studies have suggested that premotor cortices, and particularly SMA, may be involved in internal timing mechanisms, some studies targeted these cortical areas for resetting [10]. Lu et al. [3] observed that spTMS to the SMA could reset both rest PDT and postural ET, thus providing additional evidence that SMA functionally contributes to tremor generation in both disorders. They further observed that, in patients with Parkinson’s disease, the RI value elicited by M1 stimulation was significantly higher than that elicited by SMA stimulation. This pattern was not seen for ET. According to these authors, this suggests that rest PDT more strongly recruits M1 within its pathological loop. Lu et al. [3] also proposed that the relative RI values obtained from M1 versus SMA stimulation may reflect disease-specific pathophysiological differences between PDT and ET and could potentially provide a neurophysiological marker to aid in differential diagnosis in challenging cases.

## 4. Discussion

### 4.1. M1 as a convergence node for distinct tremor circuits

The major common result across the studies analyzed here is that stimulation of M1 can lead to the resetting of a wide range of rhythmic activities, such as postural ET, postural and reemergent PDT, OT (with some divergent results), palatal tremor, DT, and voluntarily mimicked rhythmic movements [2,3,5–14,17,19,32]. In contrast, rest tremor in Parkinson’s disease showed more variable resetting with M1 stimulation [3,8,9,19,28], and very little evidence of the cerebellar resetting of ET has been reported [32]. Taken together, these observations indicate that M1 functions as a final common pathway or a convergence node of multiple tremor-generating circuits rather than as a single disease-specific oscillator. This view is consistent with the suggestion of Lu et al. [3] that ET and PDT arise from different circuits (CTC vs. BGTC) but merge on M1 as a final output stage. Within this model, the ability of a tremor to reset, the speed of its resumption, and whether the reappearance latency depends on the prestimulus period can be regarded as circuit-level signs of distinct oscillators converging on a shared cortical endpoint.

### 4.2. Cerebellar involvement is tremor-type specific

The asymmetric impact of cerebellar TMS on different tremor types contributes to the understanding of the network architecture [3,5,28,29]. For PDT, cerebellar stimulation resets postural and reemergent tremor but not rest tremor [3,28], suggesting that CTC pathways may play a leading role in the postural components while rest tremor is more closely associated with BGTC loops. However in ET, cerebellar TMS did not reset tremor [3,5]. Several explanations for this were put forward in the literature, including deep and bilateral cerebellar generators that are not fully activated by unilateral surface TMS, altered afferent pathways with thalamo-cortical oscillators, and technical factors related to coil geometry and current shunting [5,27]. From a systems point of view, the simplest explanation is that cerebellar nodes are necessary but not always sufficient to maintain ET oscillations, while in postural PDT they seem to be more directly involved in the loop that generates the tremor. These differential susceptibilities to cerebellar intervention may help in refining the classification of tremors beyond clinical criteria only.

Negative or inconsistent cerebellar TMS results should be interpreted carefully. A lack of resetting does not mean that the cerebellum is not involved in the generation of tremor; rather, it could reflect the limitations of the methods of noninvasive stimulation techniques.

### 4.3. Tremor resetting, silent period, and inhibitory mechanisms

Studies of ET, PDT, and palatal tremor reported a close relationship between RI values and the TMS-induced SP [6,11,14], as well as between resetting and measures of cerebellar-brain inhibition [8]. These relationships support a model in which TMS modulates tremor primarily by transiently altering inhibitory control within key cortical or cerebellar nodes rather than by directly extinguishing the oscillatory drive.

The difference between type 0 and 1 resetting offers a helpful conceptual framework, but the RI is definitely influenced by various factors including intrinsic oscillator dynamics, SP duration, state-dependent changes in excitability, and noise in phase estimation. Interventions such as changing the mechanical load or activation level in voluntary rhythmic movement studies reveal that RI is not a pure biomarker of the oscillator itself; it is a compound index that is influenced by both network properties and the excitability state of the corticomotor system. This has important consequences for the interpretation of between-study and between-tremor type differences in RI.

### 4.4. Voluntary oscillators as a model for pathological tremors

In healthy individuals without a pathological tremor oscillator, the voluntarily mimicked tremor or voluntary rhythmic movement is generated by a sensorimotor loop that operates within the nervous system. This can be described as a “ behavioral oscillator” or a “self-paced rhythmic motor oscillator.”

The voluntarily mimicked tremor paradigms included in this review demonstrate that TMS-induced resetting is not specific to pathological oscillators. These models are important because task parameters (e.g., frequency, load, coordination mode) and cortical targets can be influenced in a systematic way, thereby allowing assessment of how SP, afferent feedback, interhemispheric coupling, and task demands influence the resetting behavior. In the study by Wagener and Colebatch [15], although the authors kept the TMS intensity constant across various load conditions, cortical excitability was highly dependent on the state and increased with mechanical load. Therefore, the same stimulation intensity does not guarantee the same physiological effect. The higher RI values at higher loads may partially be the result of load-dependent increases of M1 excitability rather than solely afferent resetting by twitch.

The similarities and differences between voluntary oscillators and pathological tremors can be enlightening [11]. For example, both of them can display fixed-latency reappearance and characteristic EMG patterns after TMS pulses. On the other hand, the dependence of reappearance latency on the prestimulus period in postural PDT but not in ET or mimicked tremor supports the possibility that some pathological oscillators have stronger intrinsic timing properties. These comparative observations emphasize the danger of interpreting any single RI value in isolation and highlight the need for considering proper physiological controls when using TMS to investigate disease mechanisms.

### 4.5. Reconciling heterogeneous findings in orthostatic and palatal tremor

The OT studies reviewed here show that small samples and slight differences in protocols can yield conflicting results. Studies that employed higher stimulus intensities and focused on contralateral leg muscles more frequently achieved resetting [7,13,17], whereas those that used lower intensities or different outcome metrics did not [31]. Rather than stating that the OT is “sometimes central” and “sometimes not,” the evidence is more compatible with a central oscillator accessible by M1-TMS under certain state and intensity conditions, and with additional contributions from pontocerebellar structures.

In cases of palatal tremor, strong resetting at high intensities, prolonged reappearance latencies, and very little impact of sensory feedback [14] strongly support a central mechanism and show that oscillatory brainstem–cerebellar loops can be easily perturbed noninvasively. Chen et al. [14] also reported that the degree of resetting was not related to the timing of the stimulus within the tremor cycle. However, this finding was constrained by only two stimulation points (burst vs. interbursts), which limits the interpretation of phase-specific effects.

The fact that palatal tremor behavior differs from that of ET, PDT, and OT in its transient slowing after TMS highlights that even when multiple tremors are resettable the detailed time course of their response may be related to syndrome-specific physiology.

## 5. Limitations

This review has several limitations. First, few human studies to date have investigated TMS-induced tremor resetting, and most of them had very small sample sizes, which makes it difficult to generalize the findings. Furthermore, there is significant methodological heterogeneity across these studies in variables such as stimulation sites, how intensity was defined, methods of analysis, EMG acquisition, and outcome metrics, all of which make it difficult to quantitatively compare the results directly. In addition, almost all of these studies were exploratory neurophysiology experiments without preregistration, and the possibility of selective reporting or unmeasured confounding factors cannot be fully excluded. It is not possible to accurately determine potential publication bias due to the limited number of studies. In addition, the literature search was restricted to English-language publications, which may have led to the omission of relevant studies published in other languages, although the overall impact of this particular limitation is likely small.

## 6. Conclusion

Tremor resetting with TMS involves the perturbation of an ongoing tremor with spTMS to infer that the stimulated structure is part of the tremor-generating and/or tremor-modulating circuit. By analyzing how the tremor reappears in terms of its phase, timing, and stability, TMS supports important inferences about the organization and connectivity of the oscillator involved.

Studies of tremor resetting using TMS collectively point to a central principle: many tremor types can be perturbed by transient cortical stimulation, but the behavior of the oscillator after this perturbation provides key circuit-level insight into the architecture of each tremor network. M1 stimulation reliably resets various types of tremor, whereas cerebellar stimulation shows tremor-specific effects, highlighting the differing contributions of the CTC and BTGC loops across tremor types. Among the tremor types, postural PDT demonstrates the clearest signature of a true central oscillator.

Despite the conceptual clarity provided by these experiments, heterogeneity in coil placement, stimulation parameters, and task conditions likely contributed to the variability across studies. Small deviations in target localization or stimulation pattern can meaningfully alter whether a TMS pulse reaches the relevant neural population, which may partly explain the inconsistent cerebellar findings reported for ET and OT. As stimulation techniques capable of precisely targeting the relevant cortical regions and the computational models designed to guide them continue to evolve, they will become essential for stimulation patterns capable of effectively engaging deep or distributed tremor circuits.

Looking ahead, closed-loop stimulation paradigms will constitute an important frontier. As the neurophysiological markers of tremor circuits become better characterized, it may become feasible to test whether real-time phase-locked stimulation/modulation can reduce stimulation load and be systematically harnessed for therapeutic purposes [36] .

Taken together, the studies in the literature to date show that TMS-induced tremor resetting provides a powerful mechanistic probe of tremor circuits, capable of distinguishing central and peripheral rhythms and even delineating how motor cortical, cerebellar, and subcortical structures exert their influence on tremor types. However, the technique remains insufficiently leveraged. By virtue of methodological refinement, deeper-target stimulation techniques, and incorporation of closed-loop and phase-locked controls, tremor resetting paradigms may ultimately evolve from a purely diagnostic method into a tool capable of guiding treatment and perhaps providing next-generation neuromodulation therapies for tremor.

## Supplementary Information

**Supplementary Table:** JBI critical appraisal checklist for analytical cross-sectional studies ratings for TMS-induced tremor resetting studies

Inclusion criteria clearly defined	JBI CRITICAL APPRAISAL CHECKLIST ITEMS							
	Subjects and the setting described	Exposure measured reliably	Standard criteria for measurement	Confounding factors identified	Strategies to deal with confounders	Outcomes measured reliably	Statistical analysis appropriate	
***Britton et al., 1993***	Y	Y	Y	Y	Y	Y	Y	Y
***Pascual-Leone et al., 1994***	Y	Y	Y	Y	Y	Y	Y	Y
***Wagener-Colebatch,1996***	Y	Y	Y	Y	Y	Y	Y	Y
***Wagener-Colebatch,1997***	Y	Y	Y	Y	Y	Y	Y	Y
***Mills-Nithi,1997***	Y	U	Y	Y	N/A	N/A	Y	Y
***Tsai et al.,1998***	Y	Y	Y	Y	N/A	N/A	Y	Y
***Manto et al.,1999***	Y	Y	Y	Y	N/A	N/A	Y	Y
***Chen et al.,2000***	Y	Y	Y	Y	Y	N/A	Y	Y
***Yu et al.,2001***	U	U	Y	Y	U	N/A	Y	Y
***Pinto et al.,2003***	Y	U	Y	Y	Y	Y	Y	Y
***Spiegel et al.,2004***	Y	Y	Y	Y	U	N/A	Y	Y
***Chen et al.,2005***	Y	Y	Y	Y	Y	Y	Y	Y
***Ni et al.,2010***	Y	Y	Y	Y	Y	Y	Y	Y
***Lu et al.,2015***	Y	Y	Y	Y	Y	Y	Y	Y
***Pattamon et al.,2016***	Y	Y	Y	Y	N/A	N/A	Y	Y
***Leodori et al.,2020***	Y	Y	Y	Y	Y	Y	Y	Y
***Helmich et al.,2021***	Y	U	Y	Y	Y	Y	Y	Y
***Leodori et al.,2022***	Y	Y	Y	Y	Y	Y	Y	Y

* JBI: Joanna Briggs Institute; Y: Yes; N: No; U: Unclear; N/A: Not Applicable

## Figures and Tables

**Figure:**
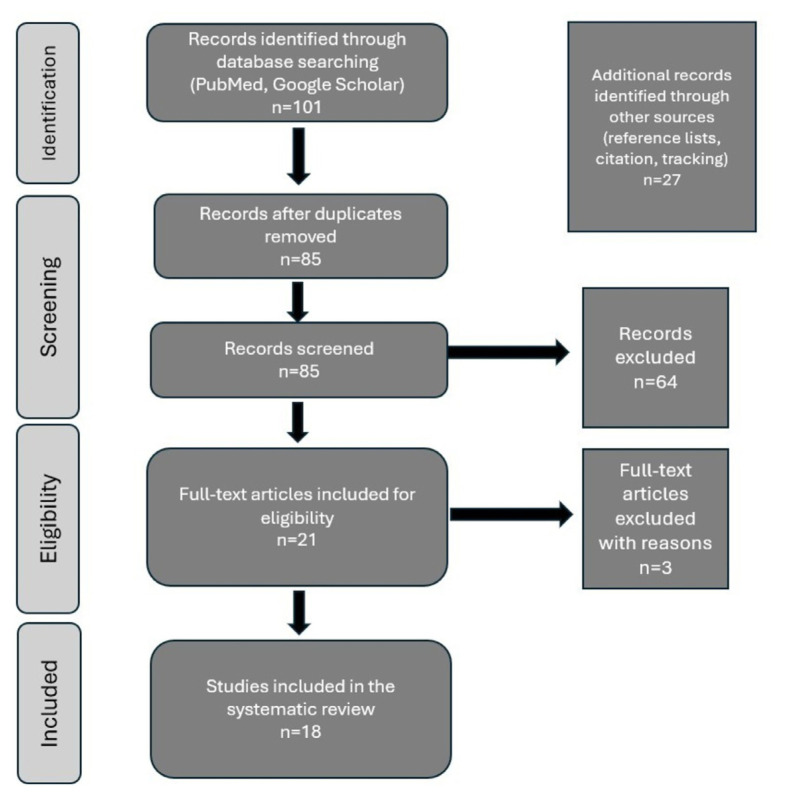
PRISMA flow diagram.

**Table 1:** Tremor resetting studies based on resetting index and using TMS. Three studies that were not included in the systematic review because they did not meet the criteria are included in this table.

Authors and year*	Tremor/movement type	Participants	Tremor/movement frequency	EMG recording muscle	TMS target	Resetting analysis method	Main findings
Britton et al., 1993 [11]	Postural PDT, ET, voluntary mimicked tremor	10 PD (ages 33–74), 12 ET (ages 25–73), 10 HA (ages 28–40)	Rest PDT: 4–6 Hz Postural PDT: 4.4–6.6 Hz ET: 4–7 Hz Mimicked tremor: 4.0–6.5 Hz	Forearm flexor and extensor muscles	Contralateral motor cortex	Resetting index	All tremors were reset PDT behaved differently compared to ET
Pascual-Leone et al., 1994 [6]	ET, postural PDT	9 ET (ages 34–61), 12 postural PDT (ages 39–69)	ET: 4.2–8.6 Hz Postural PDT: 4.3–9.1 Hz	Forearm flexor and extensor muscles bilaterally	Vertex with circular coil and contralateral motor cortex with 8-shaped coil	Resetting index	High-intensity TMS reset both ET and postural PDT Resetting depended on stimulus intensity, but was most strongly correlated with post-MEP silent period duration
Wagener and Colebatch, 1997 [15]	Voluntary rhythmic wrist movement	9 HA (2 F; ages 20–40)	Self-paced voluntary rhythmic movement	Forearm flexor and extensor muscles	Contralateral motor cortex	Resetting index	Higher extension torques yielded greater resetting index values
Tsai et al., 1998 [7]	Primary OT	2 F patients with OT (ages 58 and 68)	16 Hz	Tibialis anterior	Double cone coil positioned over the vertex	Resetting index	TMS reset OT Post-TMS period shortened initially
Manto et al., 1999 [17]	OT associated with cerebellar cortical atrophy	3 patients (2 F; ages 49, 62, 64)	14–15 Hz	Bilateral lumbar paraspinals, quadriceps femoris, tibialis anterior	TMS was applied with an angled figure-eight coil for lower limbs over vertex	Resetting index	TMS reset OT associated with cerebellar cortical atrophy
Chen et al., 2000 [14]	Symptomatic palatal tremor	5 patients (5 M; ages 67–79)	2–4 Hz	Mouth angle	Contralateral to the EMG recording with figure-eight coil	Resetting index	TMS reset symptomatic palatal tremor in all patients
Yu et al., 2001 [12]	ET and cardiac rhythm	4 patients with ET (1 F; ages 55–64), 6 HA (6 M; ages 30–65)	5–9 Hz	N/A	For hand tremor, precise coil position	Resetting index	TMS reset ET but not cardiac rhythm
Pinto et al., 2003 [5]	ET	9 patients with ET (5 F; ages 21–72), 10 HA (3 F; ages 26–81)	N/A	First dorsal interosseous, wrist flexor and extensor muscles	Cerebellar and contralateral motor cortex stimulation	Resetting index	Motor cortex stimulation reset ET but not cerebellar stimulation
Manto et al., 2003 [37]	BHFSD	Single patient (65-year-old female)	14 Hz	Flexor carpi radialis and extensor carpi radialis	TMS over the motor cortex	Resetting index	Motor cortex stimulation did not reset
Chen et al., 2005 [10]	Voluntary rhythmic index finger tapping (unimanual, bimanual in-phase, bimanual antiphase)	6 HA (1 F; ages 26–36)	1.6–4.1 Hz	Extensor digitorum communis	Motor cortex in each hemisphere	Resetting index	TMS produced intensity-dependent resetting Unimanual tasks: strong contralateral and weaker ipsilateral effects Bimanual in-phase: both hands were reset simultaneously (contralateral > ipsilateral) Bimanual antiphase: movements were resistant to resetting
Ni et al., 2010 [8]	Rest and postural PDT	10 patients with PD (1 F; mean age 62.7), 10 HA (3 F; mean age 61.1)	Rest tremor: 4.3 ± 0.9 Hz Postural tremor: 5.26 ± 0.95 Hz	Flexor carpi radialis and extensor carpi radialis	Cerebellar and contralateral M1 stimulation	Resetting index	Rest tremor was reset by M1 stimulation, but not by cerebellar stimulation Postural tremor was reset by both M1 and cerebellar stimulation
Lu et al., 2015 [3]	Rest and postural PDT, ET	10 patients with PD (4 F; mean age 62.7), 10 ET (5 F; mean age 64.3)	Rest tremor: 4–7 Hz Postural ET: 5–12 Hz	First dorsal interosseous, extensor digitorum communis, abductor pollicis brevis, abductor digiti minimi (significant tremor amplitude at the extremity of the recording muscle)	Cerebellar and contralateral M1 and SMA stimulation	Resetting index	PDT was reset by M1 and SMA stimulation but not by cerebellar stimulation Postural ET was reset by both M1 and SMA but not by cerebellar stimulation
Pattamon et al., 2016 [32]	ET and DT	12 patients with ET, 8 patients with DT	N/A	N/A	Cerebellar and contralateral M1 stimulation	Resetting index	M1 stimulation reset both ET and DT while cerebellar stimulation reset tremor in ET more than DT
Leodori et al., 2020 [9]	Rest and reemergent PDT	10 patients with PD (4 F; mean age 67)	Rest tremor: 4.27 ± 0.14 Hz Reemergent tremor: 4.74 ± 0.13 Hz	Extensor carpi radialis	Contralateral M1 stimulation	Resetting index	Both tremor types showed stable resetting
Leodori et al., 2022 [19]	Rest and postural PDT	10 PD patients with tremor suppression and 10 patients without suppression	Rest tremor: 4–5 Hz Reemergent tremor: 4–6 Hz	Extensor carpi radialis	Contralateral M1 stimulation	Resetting index	Resetting occurred for both tremor types Postural tremor resetting was stronger in patients with tremor suppression

* Studies are listed in chronological order;

** RI: resetting index; ET: essential tremor; PD: Parkinson’s disease; HA: healthy adults; M1: primary motor cortex; TMS: transcranial magnetic stimulation; F: female; M: male; OT: orthostatic tremor; EMG: electromyography; N/A: not applicable; BHFSD: bilateral high-frequency synchronous discharges; SMA: supplementary motor area; DT: dystonic tremor.

**Table 2:** Tremor resetting studies based on alternative resetting measures and using TMS. Three studies that were not included in the systematic review because they did not meet the criteria are included in this table.

Wagener and Colebatch, 1996 [2]	Voluntary rhythmic wrist movement	6 HA (3 F; ages 20–40)	2–4 Hz	Flexor carpi radialis and forearm extensor muscles	Systematic mapping of 9 cortical sites including contralateral motor cortex	Nett resetting	TMS reset the movement Confirmed the contralateral motor cortex as the effective node
Mills and Nithi, 1997 [31]	Primary OT	5 patients with OT	14–18 Hz	Leg (including hamstrings) and arm muscles	Double cone coil positioned over the vertex	Evaluated latency shift of the first post-TMS EMG burst	No evidence of tremor resetting
Pfeiffer et al., 1999 [38]**	OT	Single patient (74-year-old female)	16 Hz	N/A	N/A	N/A	Subthreshold TMS modulated timing of the tremor bursts and inhibited them at higher intensity stimulation
Wu et al., 2001 [39]	Primary OT	6 patients (3 F; ages 62–82, from Table 1)	13–17 Hz	Forearm and lower limb muscles	TMS was applied over the motor cortex	Qualitative burst realignment analysis	TMS over M1 did not reset OT Posterior fossa electrical stimulation reset OT
Spiegel et al., 2004 [13]	Primary OT	7 patients (3 F; ages 44–76)	13–18 Hz	Simultaneously both tibialis anterior muscles	Circular coil over the vertex and lumbar magnetic stimulation	Post-TMS burst timing was compared with predicted burst timing (Δt) with repeated-measures ANOVA for the correlation between time of TMS and Δt	Cortical TMS reset OT but not lumbar magnetic stimulation
Helmich et al., 2021 [28]	Rest and reemergent PDT	14 patients with PD	N/A	First dorsal interosseous, abductor pollicis brevis, flexor carpi radialis, extensor carpi radialis	Cerebellar and contralateral M1 stimulation	Tremor reset index (linear regression slope-based method)	No evidence of resetting in resting tremor Reemergent tremor was reset by cerebellar stimulation

* Studies are listed in chronological order;

** full text could not be accessed;

PD: Parkinson’s disease; HA: healthy adults; M1: primary motor cortex; TMS: transcranial magnetic stimulation; F: female; OT: orthostatic tremor; EMG: electromyography; N/A: not applicable.
